# Comparative Incidence of Urinary Tract Infections in Diabetic Women Treated with Dapagliflozin Versus Empagliflozin: A Double-Blind Randomized Clinical Trial

**DOI:** 10.5812/ijem-168824

**Published:** 2026-07-15

**Authors:** Sara Kazem-Nadi, Sara Keshtkari, Zohre Labbani-Motlagh, Vahid Mousavi

**Affiliations:** 1 Department of Internal Medicine, Faculty of Medicine, Aja University of Medical Sciences, Tehran, Iran; 2 Department of Nephrology, Faculty of Medicine, Aja University of Medical Sciences, Tehran, Iran; 3 Rajaie Cardiovascular Medical and Research Institute, Tehran, Iran; 4 Clinical Research Development Unit, Imam Reza Hospital, Aja University of Medical Sciences, Tehran, Iran; 5 Department of Endocrinology, Faculty of Medicine, Aja University of Medical Sciences, Tehran, Iran

**Keywords:** Dapagliflozin, Empagliflozin, SGLT2 Inhibitors, Type 2 Diabetes Mellitus, Urinary Tract Infections

## Abstract

**Background:**

Sodium-glucose cotransporter 2 (SGLT2) inhibitors, including dapagliflozin and empagliflozin, are widely used for glycemic control in type 2 diabetes but may affect the risk of urinary tract infection (UTI).

**Objectives:**

This study compared the incidence of UTIs among women with diabetes treated with dapagliflozin versus empagliflozin.

**Methods:**

In this randomized, double-blind clinical trial, 149 diabetic women aged ≥ 18 years were assigned to receive dapagliflozin 5 mg (n = 79) or empagliflozin 10 mg (n = 70) daily for three months. Fasting blood sugar (FBS), glycated hemoglobin (HbA1c), and serum creatinine (Cr) were measured at baseline and after treatment. Urinalysis and urine culture were used to confirm UTIs. Data were analyzed using SPSS version 28.0, with P < 0.05 considered statistically significant.

**Results:**

The mean age was 62.7 ± 9.7 years, with no baseline differences between groups. UTIs occurred in 12 patients overall: 2 (2.5%) in the dapagliflozin group and 10 (14.3%) in the empagliflozin group (OR = 0.16; 95% CI, 0.03 - 0.74; P = 0.009). No significant intergroup differences were observed in FBS, HbA1c, or Cr levels.

**Conclusions:**

Empagliflozin was associated with a higher short-term incidence of UTIs than dapagliflozin, despite similar metabolic outcomes. Monitoring for UTIs is recommended when prescribing SGLT2 inhibitors, particularly empagliflozin. Trial Registration: This trial is registered with the Iranian Registry of Clinical Trials (IRCT20250129064554N1).

## 1. Background

Diabetes mellitus (DM) is a major public health concern in Iran, with a steadily increasing prevalence over the past few decades. A comprehensive analysis of studies conducted between 1996 and 2023 showed that type 2 diabetes affects approximately 13.4% of women and 10.8% of men in Iran, with higher prevalence rates in older age groups (1). Given the growing burden of diabetes, disease-related complications, including urinary tract infections (UTIs), have become a critical focus. UTIs are particularly common among patients with diabetes, with studies from Iran reporting a high incidence of both symptomatic and asymptomatic infections. For example, a study involving 500 patients with diabetes found that 37.2% had UTIs, with uncontrolled glycemia significantly increasing the risk (2). Similarly, another Iranian study reported asymptomatic UTIs in 16.9% of patients with diabetes, with women disproportionately affected compared with men. These findings underscore the importance of addressing UTIs as a common and potentially severe complication in diabetic populations.

Sodium-glucose cotransporter 2 (SGLT2) inhibitors, such as empagliflozin and dapagliflozin, have emerged as valuable therapeutic options for managing type 2 diabetes because they improve glycemic control, reduce body weight, and lower blood pressure (3). These benefits have made SGLT2 inhibitors an important component of diabetes management. However, their use has also been associated with potential adverse effects, particularly an increased risk of urogenital infections (4).

As of 2025, research indicates that urogenital symptoms led to treatment discontinuation in 40% of male patients who stopped SGLT2 inhibitor therapy, suggesting that these adverse effects may influence treatment adherence and outcomes (4). Recent systematic reviews and meta-analyses have provided further insight into this association. For instance, an analysis of landmark trials (5), including CANVAS (6), CREDENCE (7), DECLARE-TIMI 58 (8), and EMPA-REG (9), published in 2024, demonstrated that SGLT2 inhibitor use is associated with a slightly increased risk of UTIs compared with placebo, although the overall incidence remains low (5). Similarly, another review conducted in 2023 reported that dapagliflozin, particularly at higher doses and with longer treatment durations, may confer a greater risk of UTIs than placebo or other active treatments (10). These findings suggest that although SGLT2 inhibitors may not increase UTI risk across all agents in the class, specific drugs and dosing regimens may warrant closer monitoring.

The association between diabetes and recurrent UTIs has also been examined in recent studies. A 2023 study found that women with DM are at higher risk of recurrent UTIs than women without diabetes, with *E. coli* as the predominant pathogen (11). This increased susceptibility highlights the importance of understanding how SGLT2 inhibitors, which are widely prescribed for glycemic control, may influence UTI rates in diabetic populations. However, findings on the overall safety of SGLT2 inhibitors with respect to UTIs remain mixed. For example, a 2021 systematic review of 55 studies involving 29,574 patients concluded that SGLT2 inhibitors were not significantly associated with an increased risk of UTIs compared with other antidiabetic medications (12). However, an earlier investigation emphasized that certain SGLT2 inhibitors, particularly dapagliflozin and canagliflozin, may still increase the risk of UTIs, especially in combination therapies or in patients with specific comorbidities (13).

Despite these advances, a notable gap remains in research examining the incidence of UTIs after SGLT2 inhibitor treatment among women with diabetes in Iran. Regional variations in patient demographics may influence UTI incidence and outcomes, making local data essential. Furthermore, women are disproportionately affected by UTIs (14), and the intersection of diabetes and SGLT2 inhibitor therapy in this population warrants targeted investigation.

## 2. Objectives

This randomized controlled clinical trial was designed to compare the incidence of UTIs in diabetic women receiving either empagliflozin 10 mg daily or dapagliflozin 5 mg daily. By focusing on Iranian female patients, this study aimed to generate clinically relevant evidence to inform guidelines and improve patient care in this underserved population. Clarifying the association between SGLT2 inhibitors and UTIs in this setting is essential for optimizing treatment strategies while minimizing adverse effects.

## 3. Methods

### 3.1. Study Design and Setting

This randomized, double-blind clinical trial was conducted at Imam Reza Teaching Hospital, Tehran, Iran. Patient recruitment was performed from March 2025 to August 2025. Participants were randomly assigned to one of two treatment groups: Dapagliflozin 5 mg daily or empagliflozin 10 mg daily. Randomization was performed using a computer-generated random sequence, implemented using sealed envelopes and kept by one of the authors, who was not involved in enrollment, data collection, or patient assessment. Simple randomization was applied. Allocation concealment was maintained using pharmacy-controlled allocation. Participants were enrolled, and treatment assignment was performed. Patients, treating physicians, laboratory personnel, outcome assessors, and data analysts remained blinded throughout the study period. Urine culture interpretation was performed without knowledge of treatment allocation.

### 3.2. Participants and Eligibility Criteria

Type 2 diabetes mellitus was diagnosed according to [ADA 2024 criteria or applicable guideline], including fasting plasma glucose ≥ 126 mg/dL, HbA1c ≥ 6.5%, 2-hour plasma glucose ≥ 200 mg/dL during oral glucose tolerance testing, or use of antidiabetic medications. Women aged ≥ 18 years with a confirmed diagnosis of type 2 diabetes and a stable clinical condition were eligible for inclusion. Participants were required to provide written informed consent and agree to comply with the study protocol. Exclusion criteria included known intolerance to dapagliflozin or empagliflozin, pregnancy or intention to become pregnant, severe renal impairment (eGFR < 25 mL/min/1.73 m^2^), previous bariatric surgery, or a history of urinary tract infection. Participants were withdrawn if they declined further participation, were lost to follow-up, or developed an acute illness necessitating treatment discontinuation. Eligible patients were recruited through convenience sampling from women with diabetes attending the hospital. Baseline variables associated with UTI risk, including age, glycemic control, renal function, previous UTI history, menopausal status, and concomitant medications, were recorded.

### 3.3. Sample Size Calculation

At the time of study design, reliable data directly comparing the incidence of UTIs between dapagliflozin and empagliflozin in women with type 2 diabetes were unavailable. Therefore, a precise effect size for a formal superiority sample size calculation could not be established. Consequently, this study was designed as an exploratory randomized clinical trial intended to generate preliminary comparative data and provide estimates of event rates for future confirmatory studies.

The target sample size was determined based on feasibility considerations, including the expected number of eligible patients presenting to the study center during the predefined recruitment period and the resources available for this investigator-initiated academic study. Enrollment continued throughout the recruitment period, resulting in approximately 70 participants per treatment group. This sample size was considered adequate to obtain preliminary estimates of treatment-associated UTI incidence and to support the planning of future adequately powered multicenter trials. Accordingly, the findings should be interpreted as exploratory and hypothesis-generating.

### 3.4. Interventions

To preserve blinding, study medications were repackaged into identical containers and labeled using participant codes only. Packaging and labeling were performed.

Participants randomized to the dapagliflozin group received dapagliflozin 5 mg orally once daily, whereas those randomized to the empagliflozin group received empagliflozin 10 mg orally once daily for three months. Participants continued their routine diabetes care and concomitant medications as prescribed by their treating physicians. No study-mandated dietary modifications, lifestyle interventions, or additional therapeutic measures were introduced during the study period.

### 3.5. Data Collection and Outcome Measures

At the end of the three-month treatment period, biochemical parameters, including blood sugar (BS), fasting blood sugar (FBS), glycated hemoglobin (HbA1c), and serum creatinine (Cr), were measured. Urine analysis (UA) and urine culture (UC) were performed to identify UTIs. Pyuria categories were defined according to urine white blood cell (WBC) count: normal (0 - 5 WBC/HPF), moderate (6 - 10 WBC/HPF), and high (> 10 WBC/HPF). These categories were selected based on conventional laboratory interpretation of inflammatory urinary findings.

The primary outcome was the incidence of UTIs. UTI was defined as the presence of urinary symptoms, including dysuria, urinary frequency, urinary urgency, suprapubic pain, or fever attributable to urinary infection, together with a positive urine culture showing ≥ 10^5^ CFU/mL of a urinary pathogen from a properly collected midstream clean-catch specimen. Samples meeting predefined contamination criteria were repeated. Urine cultures were obtained only at the three-month visit. Antibiotic exposure during follow-up was recorded and considered during outcome assessment. Symptomatic culture-negative findings and asymptomatic pyuria were not considered diagnostic. Secondary outcomes included changes in biochemical parameters (BS, FBS, HbA1c, and Cr) after three months of treatment.

### 3.6. Statistical Analysis

Statistical analyses were performed using IBM SPSS Statistics version 28.0. Data normality was assessed using the Shapiro-Wilk test. Continuous variables are presented as mean ± standard deviation or median (interquartile range), as appropriate, and were compared between groups using the independent-samples t-test or the Mann-Whitney U test. Categorical variables were analyzed using the chi-square test or Fisher's exact test, as appropriate.

The primary outcome, culture-confirmed UTI within three months, was compared using Fisher's exact test, and the effect size was reported as an odds ratio (OR) with a 95% confidence interval (CI), using dapagliflozin as the reference group.

Longitudinal outcomes (FBS, HbA1c, and creatinine) were analyzed using within-group paired tests and between-group comparisons at each time point. In addition, overall treatment effects over time were assessed using group × time interaction analysis.

Correlations were evaluated using Pearson's or Spearman's correlation coefficients, as appropriate. All tests were two-sided, and P < 0.05 was considered statistically significant. Missing data were handled using complete-case analysis.

### 3.7. Ethical Considerations

The study protocol was approved by the institutional ethics committee (Approval ID: IR.AJAUMS.REC.1403.153), and the study protocol was registered (IRCT20250129064554N1).

Written informed consent was obtained from all participants before enrollment and before any study-related procedures were performed. The trial was conducted in accordance with the principles of the Declaration of Helsinki. Participants were informed of their right to withdraw from the study at any time without penalty.

## 4. Results

### 4.1. Demographic Characteristics

A total of 196 patients were assessed for eligibility. Thirty-nine patients were excluded before randomization because they did not meet the inclusion criteria or declined to participate. Seventy-three patients were allocated to empagliflozin, and 84 were allocated to dapagliflozin. During follow-up, 7 patients were lost to follow-up, and 1 patient discontinued treatment. Therefore, only participants who completed the three-month follow-up and had complete outcome data were included in the final analysis.

**Figure 1.**
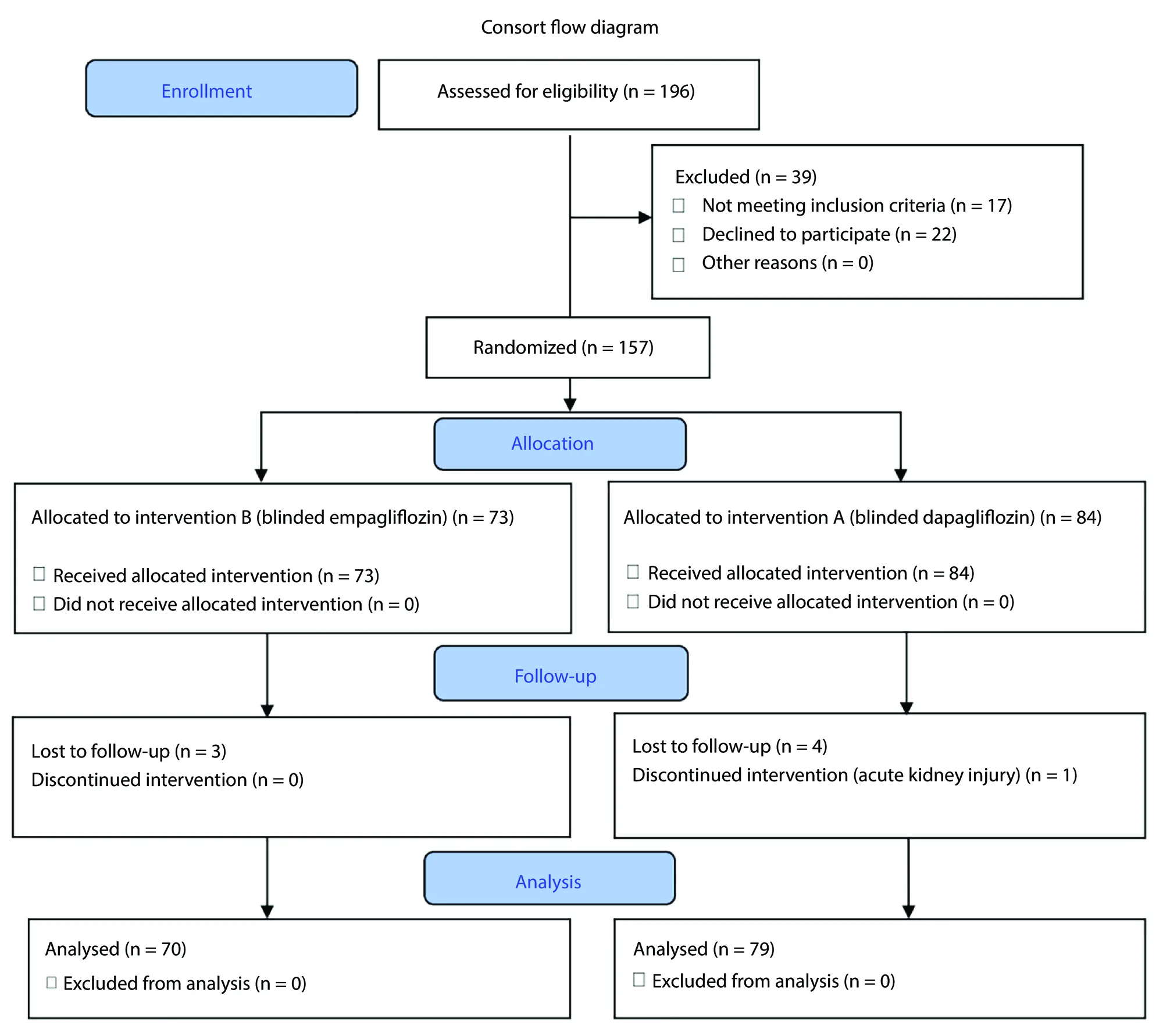
CONSORT flow diagram

The study was completed by 79 patients in the dapagliflozin group and 70 patients in the empagliflozin group. The CONSORT flow diagram is presented in Figure 1. The mean age was 62.30 ± 10.40 years in the dapagliflozin group and 63.10 ± 9.00 years in the empagliflozin group. HbA1c followed a normal distribution, whereas fasting blood glucose, random blood glucose, and serum creatinine deviated from normality. No statistically significant differences were observed in baseline parameters (Table 1).

**Table 1.** Comparison of Age and Serum Markers Between Dapagliflozin and Empagliflozin Treatment Groups ^a^

Variables	Dapagliflozin (n = 79)	Empagliflozin (n = 70)	Between-Group P Value (Baseline)	Within-Group Change P Value	Group × Time Interaction P Value
**Age (y)**	62.30 ± 10.40	63.10 ± 9.00	0.998	—	0.912
**FBS (mg/dL)**					
Baseline	146 (130 - 160)	140 (125 - 153)	0.178	—	—
Post	125 (108 - 138)	116 (103 - 130)	—	< 0.001	0.041
**BS (mg/dL)**					
Baseline	187 (153 - 205)	190 (160 - 217)	0.106	—	—
Post	159 (132 - 194)	155 (127 - 182)	—	< 0.001	0.038
**HbA1c (%)**					
Baseline	7.6 ± 0.6	7.2 ± 0.8	0.062	—	—
Post	6.72 ± 0.80	6.50 ± 0.60	—	< 0.001	0.087
**Creatinine (mg/dL)**					
Baseline	0.9 (0.7 - 1.0)	0.9 (0.8 - 1.0)	0.505	—	—
Post	0.9 (0.8 - 1.0)	0.9 (0.8 - 1.0)	—	0.214	0.505

^a^ Values are expressed as mean ± SD or median (interquartile range [IQR]), as appropriate. Between-group comparisons at baseline were performed using the independent-samples t test or Mann-Whitney U test. Within-group comparisons (baseline vs follow-up) were performed using the paired t test or Wilcoxon signed-rank test, as appropriate. Overall treatment effects over time were assessed using group × time interaction analysis. All tests were two-sided, and P < 0.05 was considered statistically significant. Abbreviations: BS, blood sugar; Cr, serum creatinine; FBS, fasting blood sugar; HbA1c, hemoglobin A1c; IQR, interquartile range; SD, standard deviation.

### 4.2. Primary Outcome

#### 4.2.1. Incidence of UTI Between Groups

**Figure 2.**
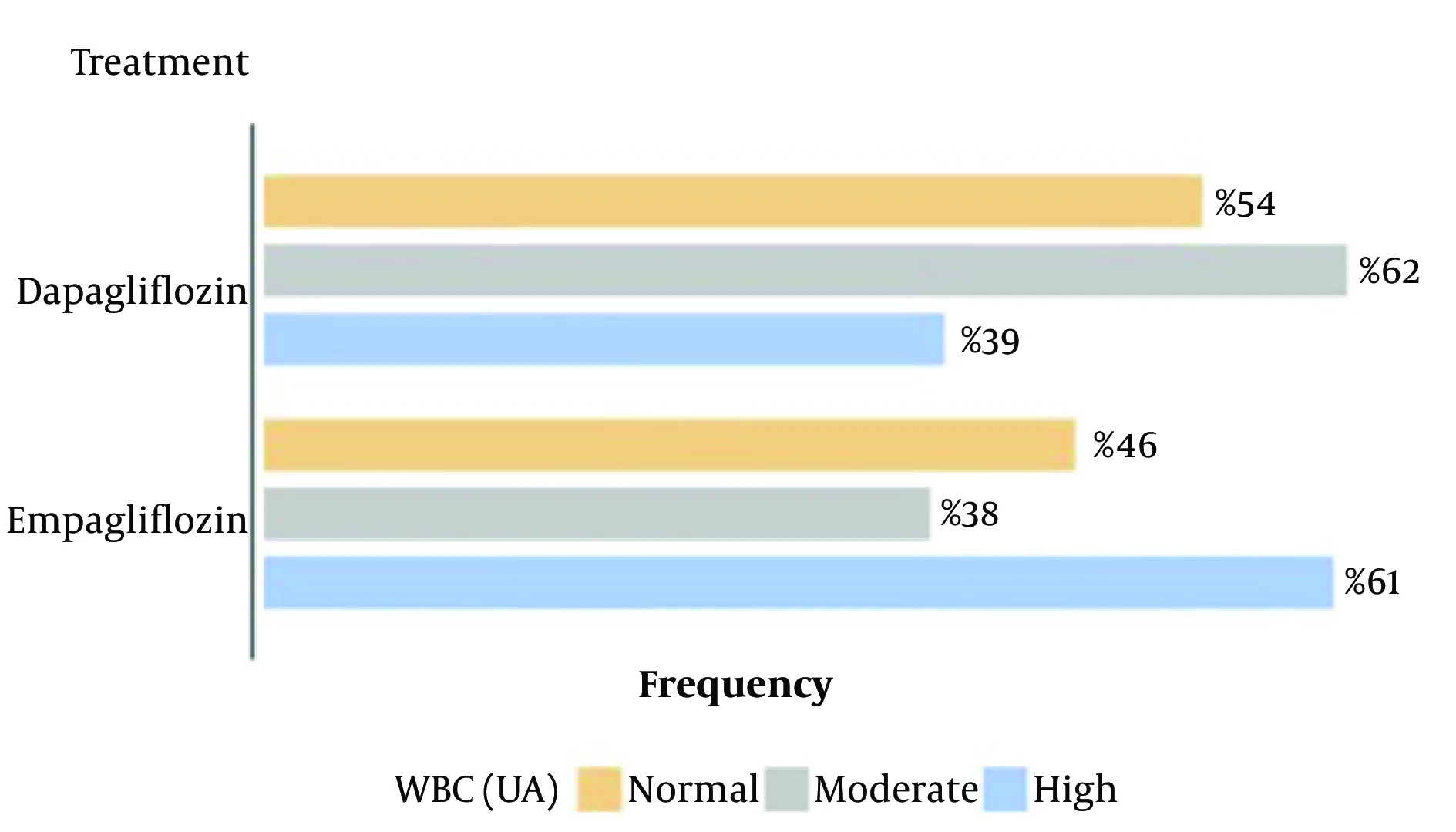
Distribution of normal, moderate, and high white blood cell counts in urine analysis test results in the dapagliflozin and empagliflozin treatment groups.

By the end of the three-month treatment period, 12 patients had developed UTIs. The incidence of positive urine cultures was significantly higher in the empagliflozin group than in the dapagliflozin group (14.3% vs 2.5%), corresponding to an odds ratio of 0.16 (2 [2.5%] in the dapagliflozin group and 10 [14.3%] in the empagliflozin group). As presented in Table 2, patients with positive urine cultures were slightly older than those with negative cultures (64.10 ± 11.60 vs 62.60 ± 9.60 years), although the difference was not statistically significant (Figure 2). Similarly, no significant differences were observed between the groups with respect to serum markers.

**Table 2.** Comparison of Age and Serum Markers According to Urine Culture Results and Urine Analysis White Blood Cell Count ^a^

Variables	Urine Culture			UA WBC			
	Positive (n = 12)	Negative (n = 137)	P Value	Normal (n = 110)	Moderate (n = 21)	High (n = 18)	P Value
**Age (y)**	64.10 ± 11.60	62.60 ± 9.60	0.474	62.30 ± 9.50	63.90 ± 10.00	63.70 ± 10.80	0.690
**FBS (mg/dL)**	118.00 ± 35.32	123.80 ± 23.87	0.187	122.12 ± 24.14	129.76 ± 31.31	123.22 ± 20.91	0.438
**BS (mg/dL)**	172.17 ± 37.87	164.23 ± 51.26	0.267	161.95 ± 50.15	174.76 ± 58.96	171.11 ± 39.52	0.485
**HbA1c (%)**	6.53 ± 0.80	6.62 ± 0.71	0.693	6.64 ± 0.74	6.54 ± 0.61	6.53 ± 0.68	0.733
**Cr (mg/dL)**	0.89 ± 0.21	0.92 ± 0.18	0.708	0.92 ± 0.18	0.87 ± 0.16	0.94 ± 0.20	0.408

^a^ Values are expressed as mean ± SD. Statistical analyses were performed based on the distribution of each variable. P < 0.05 was considered statistically significant. Abbreviations: BS, blood sugar; Cr, serum creatinine; FBS, fasting blood sugar; HbA1c, hemoglobin A1c; SD, standard deviation.

Table 3 summarizes the distribution of UA and UC findings across the two treatment groups. Although a higher frequency of elevated WBC counts in UA was observed in the empagliflozin group, this difference was not statistically significant.

**Table 3.** Frequency of Urine Analysis White Blood Cell and Urine Culture Categories Among Treatment Groups ^a^

Outcomes	Dapagliflozin (n = 79); (%)	Empagliflozin (n = 70); (%)	Odds Ratio	P Value
**UA WBC Range**				
Normal	59 (74.7)	51 (72.9)	—	0.346
Moderate	13 (16.5)	8 (11.4)	—	0.346
High	7 (8.8)	11 (15.7)	—	0.346
**UC**				
Positive	2 (2.5)	10 (14.3)	0.16 [0.03, 0.74]	0.009
Negative	77 (97.5)	60 (85.7)	0.16 [0.03, 0.74]	0.009

^a^ White blood cell ranges in the urine analysis were defined as 0 to 5, 6 to 10, and more than 10 leukocytes for normal, moderate, and high categories, respectively. P < 0.05 was considered statistically significant. The odds ratio represents dapagliflozin relative to empagliflozin. Abbreviations: UA, urine analysis; UC, urine culture; WBC, white blood cell.

### 4.3. Secondary Outcomes

#### 4.3.1. Blood Glucose Profile Between Groups

At three months, BS, FBS, and HbA1c were 159 versus 155 mg/dL, 125 versus 116 mg/dL, and 6.72% versus 6.50% in the dapagliflozin and empagliflozin groups, respectively. No statistically significant differences were observed between groups for any glycemic parameter (Table 1).

#### 4.3.2. Effect of Treatment on Renal Function

Serum creatinine was 0.9 mg/dL in both groups at baseline and remained 0.9 mg/dL at three months in both the dapagliflozin and empagliflozin groups, with no statistically significant between-group or within-group differences (Table 1).

## 5. Discussion

The study found a significant difference in UTI incidence between diabetic women treated with dapagliflozin and those receiving empagliflozin. Over three months, UTIs occurred in 2.5% of the dapagliflozin group and 14.3% of the empagliflozin group (OR = 0.16), indicating a higher UTI risk with empagliflozin. These results support differential infection risk among SGLT2 inhibitors.

A 2024 Turkish study found no significant association between asymptomatic pyuria or bacteriuria and subsequent UTI development in women with type 2 diabetes initiating SGLT2 inhibitors, suggesting that baseline urinary abnormalities are unreliable predictors of infection (15). Although our study did not assess these markers, the observed UTI rates underscore the need to monitor for symptomatic infections, particularly in diabetic populations (16), in which prevalence may vary regionally because of health care practices, hygiene, and microbial ecology.

Conversely, a US retrospective study of male veterans using empagliflozin with urinary catheters reported a slight, nonsignificant decrease in UTI frequency (0.09 vs 0.07 UTIs/month) (17). Although this population differs from ours, these findings suggest that empagliflozin may not inherently increase UTI risk. In contrast, our results indicate a higher UTI incidence in women, suggesting that sex-specific factors may influence susceptibility to infection during empagliflozin therapy.

A Romanian cross-sectional study supports our findings that female sex is a predisposing factor for UTIs. Among 328 patients with type 2 diabetes, women were more likely to develop UTIs regardless of medication, and elevated HbA1c was an additional risk factor (18). These results align with our observation of higher fasting blood sugar and HbA1c in the moderate WBC group, emphasizing that glycemic control and overall metabolic health are important considerations when prescribing SGLT2 inhibitors.

A longitudinal Chinese study identified advanced age, poor glycemic control, and impaired renal function as risk factors for first-time UTIs in diabetic patients using SGLT2 inhibitors (19). Although our cohort had a similar mean age, we did not find a significant association between serum creatinine and UTI incidence. However, the slightly higher creatinine levels observed in patients with elevated WBC counts suggest that renal function warrants further investigation as a potential biomarker for infection risk.

Two additional studies examined dapagliflozin and UTI incidence. A Syrian study of 108 patients reported no significant increase in UTIs, although genital infections were more common (20). Similarly, a retrospective cohort study conducted in the Philippines involving 253 diabetic patients found that poor glycemic control strongly predicted genitourinary infections, with three- and six-month UTI incidences of 2.37% and 21.78%, respectively, aligning with the rates observed in our dapagliflozin group (21). These findings suggest that dapagliflozin has a relatively low impact on UTI risk.

Pooled analyses provide broader insights into the comparative UTI safety of SGLT2 inhibitors. Consistent with our findings, a pooled analysis of noninterventional cohorts from the United Kingdom, United States, and Medicare databases showed that dapagliflozin initiators had a lower incidence of serious UTIs than users of other glucose-lowering drugs, with adjusted incidence rate ratios of 0.76 in females and 0.74 in males (22). Similarly, a pooled analysis of 12 randomized placebo-controlled trials reported UTI rates of 3.6% to 5.7% in dapagliflozin-treated patients versus 3.7% in placebo-treated patients, with no dose-dependent increase (23). These findings are consistent with our observation of a lower frequency of culture-confirmed UTIs among dapagliflozin-treated participants; however, direct comparative evidence remains limited, and larger studies are needed to confirm whether a true difference exists between agents. Overall, the available evidence does not establish a definitive hierarchy of UTI risk among SGLT2 inhibitors, although some studies, including the present trial, suggest the possibility of differential risk that warrants further investigation.

The biological basis for the observed difference in UTI incidence between empagliflozin and dapagliflozin remains unclear. Although all SGLT2 inhibitors promote urinary glucose excretion and may thereby facilitate bacterial growth within the urinary tract, current evidence does not establish a consistent relationship between the degree of glucosuria and UTI risk. Differences in pharmacokinetic properties, SGLT2:SGLT1 selectivity, urinary glucose exposure, renal function, host immunity, and urinary microbiota may theoretically contribute to variation in infection susceptibility (24, 25). However, no mechanistic study has definitively demonstrated a higher UTI risk with empagliflozin than with dapagliflozin. Therefore, the observed difference should be interpreted cautiously and may reflect a combination of pharmacologic, patient-related, and random factors. Further mechanistic and multicenter clinical studies are required to clarify whether a true drug-specific difference exists.

### 5.1. Study Limitations

This study provides insights into comparative UTI risks among women with type 2 diabetes mellitus in Iran; however, several limitations should be acknowledged. First, this was a single-center study with a relatively modest sample size and only 12 culture-confirmed UTI events. Second, the study included women only; therefore, the findings may not be generalizable to men. Third, follow-up was limited to three months and may not reflect long-term infection risk. Fourth, only participants who completed follow-up and had available outcome data were included in the final analysis, which may have introduced attrition bias. Consequently, the findings should be interpreted cautiously until confirmed by larger multicenter randomized trials.

### 5.2. Conclusions

In this randomized clinical trial, empagliflozin-treated patients experienced a higher frequency of culture-confirmed UTIs than dapagliflozin-treated patients during three months of follow-up. However, given the single-center design, limited sample size, and small number of UTI events, these findings should be considered preliminary. Larger multicenter studies are needed to confirm whether clinically relevant differences in UTI risk exist among individual SGLT2 inhibitors.

Despite these limitations, this study provides clinically meaningful evidence that dapagliflozin may pose a lower UTI risk than empagliflozin among diabetic women and highlights the need for careful monitoring when prescribing SGLT2 inhibitors, particularly empagliflozin, in diabetic women. Future research with larger, multicenter cohorts and extended follow-up periods is necessary to validate these findings and further explore the underlying mechanisms contributing to UTI risk. Such efforts will help refine treatment strategies and improve patient outcomes in diabetes care by enhancing precision in pharmacotherapy and safety monitoring.

## Data Availability

The dataset presented in the study is available on request from the corresponding author during submission or after publication. The data are not publicly available due to ethical restrictions and patient confidentiality.

## Clinical Trial Registration Code:

The trial is registered at Iranian Registry of Clinical Trials (IRCT20250129064554N1).
